# Supplementary material for: Environmental Effects on Taxonomic Turnover in Soil Fauna across Multiple Forest Ecosystems in East Asia
Source: Insects. 2022 Nov 30;13(12):1103. doi: 10.3390/insects13121103 (PMC9786105; doi:10.3390/insects13121103)
Supplement: Supplementary file 1 [file insects-13-01103-s001.zip › insects-2001240-supplementary.pdf]

Environmental Effects of Soil Fauna on Taxonomic Turnover across Multiple Forest Ecosystems in East Asia

Table S1. Summary of the soil animal orders composition in the 14 sites in East Asia, where TH = Tahe, ARS = Aershan, CBS = Changbaishan, DLS = Donglingshan, BTM = Baotianman, BDGS = Badagongshan, TTS = Tinatongshan, SMT = Shimentai, JFL = Jianfengling, XSBN = Xishuangbanna, GH = Guanghua, SPK = Sapporo, HMA = Hiroshima, and YS = Yushan. Sap, Omn, Phy, and Pre represent saprozoic, omnivores, phytophage, and predacity, respectively. “+” represents orders appear in the site. “-” represents species do not appear in the site.

Order Name	Code	Traits	TH	ARS	CBS	DLS	BTM	BDGS	TTS	SMT	JFL	XSBN	GH	SPK	HMA	YS
Acarina	Aca	Sap	+	+	+	+	+	+	+	+	+	+	+	+	+	+
Mesostigmata	Mesos	Pre	+	+	+	+	+	+	+	+	+	+	+	+	+	+
Collembola	Coll	Omn	+	+	+	+	+	+	+	+	+	+	+	+	+	+
Diptera	Dipt	Omn	+	+	+	+	+	+	+	+	+	+	+	+	+	+
Tubificida	Tub	Sap	+	+	+	+	+	-	+	+	+	+	-	+	-	+
Coleoptera	Cole	Pre	+	+	+	+	+	+	+	+	+	+	+	+	+	+
Geophilomorpha	Geo	Pre	+	+	+	+	+	+	+	+	+	+	+	+	+	+
Juliformia	Julif	Sap	+	-	+	-	+	+	+	-	+	-	+	-	-	+
Homoptera	Hom	Phy	+	+	+	+	-	-	+	+	+	-	+	+	-	+
Thysanoptera	Thy	Phy	+	+	+	+	+	+	+	+	+	+	+	-	+	-
Hymenoptera	Hym	Omn	+	+	+	+	+	+	+	+	+	+	+	+	+	+
Lithobiomorpha	Lit	Pre	+	+	+	+	+	+	+	+	+	+	+	-	+	+
Protura	Pro	Sap	+	-	-	-	-	+	+	-	+	+	+	-	+	+
Araneae	Ara	Pre	+	+	+	+	+	+	+	+	+	+	+	+	+	+
Lepidoptera	Lep	Phy	+	+	+	+	+	+	+	+	+	+	+	+	+	+
Hemiptera	Hem	Omn	+	+	+	+	+	+	+	+	+	-	+	+	+	+
Rhabditida	Rha	Phy	+	+	-	-	+	-	+	-	+	+	-	-	-	+
Opiliones	Opi	Pre	+	-	+	+	-	+	+	-	-	+	-	-	+	-
Lumbricida	Lum	Sap	+	-	+	+	+	-	-	-	-	-	-	+	+	-
Amphipoda	Amp	Sap	+	-	-	-	-	-	-	-	-	-	-	+	+	-
Symphyla	Sym	Sap	+	-	-	+	-	+	+	-	+	+	+	-	+	+
Pseudoscorpiones	Pse	Pre	-	-	+	+	+	+	+	+	+	+	+	+	+	+
Diplura	Dipl	Phy	-	-	+	+	+	+	+	+	+	+	+	-	+	+
Glomerida	Glo	Sap	-	-	-	+	-	-	-	-	-	+	-	-	+	-
Spirobolida	Spi	Sap	-	-	-	+	-	-	-	-	-	+	-	-	-	-
Stylommatophora	Sty	Omn	-	-	-	+	-	-	-	-	-	-	-	-	-	-
Mesogastropoda	Mes	Phy	-	-	-	+	-	-	-	+	+	-	-	+	+	-
Dermaptera	Der	Omn	-	-	-	+	-	+	-	-	+	+	-	+	-	-
Neuroptera	Neu	Pre	-	-	-	+	-	-	-	+	-	-	-	-	-	-
Orthoptera	Ort	Phy	-	-	-	+	+	+	+	-	+	+	+	+	-	+
Psocoptera	Pso	Phy	-	-	-	+	-	+	+	+	+	-	+	-	-	+
Isopoda	Isopo	Omn	-	-	-	+	+	+	+	+	+	-	+	-	+	-

Eosentomata	Eos	Sap	-	-	-	+	+	-	-	-	-	-	-	-	-	-
Sinentomata	Sin	Sap	-	-	-	-	+	-	-	-	-	-	-	-	-	-
Scolopendromorpha	Sco	Pre	-	-	-	-	+	+	+	+	+	-	-	-	+	+
Polyxenida	Polyx	Sap	-	-	-	-	+	-	-	-	-	-	+	-	-	-
Archaeogastropoda	Arc	Omn	-	-	-	-	+	-	-	-	-	-	-	-	-	-
Blattaria	Bla	Omn	-	-	-	-	-	+	+	+	+	-	+	-	-	-
Isoptera	Isopt	Pre	-	-	-	-	-	+	+	-	+	+	+	-	+	-
Microcoryphia	Mic	Phy	-	-	-	-	-	+	-	-	+	-	-	-	-	-
Harpacticoida	Har	Omn	-	-	-	-	-	+	-	-	+	-	-	-	-	-
Tetramerocerata	Tet	Sap	-	-	-	-	-	+	+	-	+	+	-	-	+	-
Polydesmida	Polyd	Sap	-	-	-	-	-	-	-	+	-	-	-	-	+	-
Scutigeromorpha	Scu	Pre	-	-	-	-	-	-	-	+	+	-	+	-	-	-
Schizomida	Sch	Sap	-	-	-	-	-	-	-	-	+	-	-	-	-	-
Plecoptera	Ple	Phy	-	-	-	-	-	-	-	-	+	-	-	-	-	-
Sphaerotheriida	Sph	Sap	-	-	-	-	-	-	-	-	-	+	-	-	-	-
Thysanura	Thysanura	Phy	-	-	-	-	-	-	-	-	-	-	+	+	+	+
Chordeumatida	Cho	Sap	-	-	-	-	-	-	-	-	-	-	-	+	+	-
Julida	Julid	Sap	-	-	-	-	-	-	-	-	-	-	-	-	+	-

Table S2. The iNEXT numerical values for the three special cases of q = 0, 1 and 2 for soil fauna orders in East Asia [62].

Step 1. Sample completeness profiles (panel a in Figure 2)			
Completeness	q=0	q=1	q=2
Overall	93.1%	97.9%	99.6%
Subtropics	93.5%	95.1%	96.7%
Temperate zone	87.2%	94.8%	98.7%
Tropics	85.9%	90.3%	94.5%
**Step 2. Asymptotic analysis (panels b and c in Figure 2)**			
Diversity	q=0	q=1	q=2
Overall			
Asymptotic	53.71	40.18	34.32
Empirical	50.00	38.16	33.26
Undetected	3.71	2.02	1.06
Subtropics			
Asymptotic	36.37	34.59	32.94
Empirical	34.00	31.63	30.10
Undetected	2.37	2.96	2.84
Temperate zone			
Asymptotic	51.62	39.55	33.05
Empirical	45.00	35.80	31.10
Undetected	6.62	3.75	1.95
Tropics			
Asymptotic	44.26	40.13	36.50
Empirical	38.00	34.61	32.34
Undetected	6.26	5.52	4.16
**Step 3. Non-asymptotic coverage-based rarefaction and extrapolation (panel d in Figure 2) Maximum standardized coverage C_{max} = 0.98**			
Diversity	q=0	q=1	q=2
Overall	50.15	38.20	33.28
Subtropics	35.40	32.87	30.85
Temperate zone	49.05	37.62	31.83
Tropics	42.97	38.43	34.29
**Step 4: Evenness among orders abundances (panel e)**			
Evenness	Pielou *J’*	q=1	q=2
Overall	0.93	0.76	0.66
Subtropics	0.98	0.93	0.87
Temperate zone	0.93	0.76	0.64
Tropics	0.97	0.89	0.79

Table S3. The iNEXT numerical values for soil fauna of sample completeness profiles and asymptotic analysis in East Asia.

Step 1. Sample completeness profiles

Completeness	q=0	q=1	q=2
TH	76.0%	100.0%	100.0%
ARS	100.0%	100.0%	100.0%
SPK	75.0%	100.0%	100.0%
CBS	83.0%	100.0%	100.0%
DLS	89.0%	100.0%	100.0%
GH	99.0%	100.0%	100.0%
HMA	90.0%	100.0%	100.0%
BTM	95.0%	100.0%	100.0%
TTS	94.0%	100.0%	100.0%
BDGS	76.0%	100.0%	100.0%
SMT	88.0%	100.0%	100.0%
YS	82.0%	100.0%	100.0%
XSBN	89.0%	100.0%	100.0%
JFL	93.0%	100.0%	100.0%

Step 2. Asymptotic analysis

Diversity	q=0	q=1	q=2	q=0	q=1	q=2	q=0	q=1	q=2
	TH			GH			SMT		
Asymptotic	80.60	7.90	5.38	27.17	3.54	2.56	100.50	4.62	2.22
Empirical	61.00	7.88	5.37	27.00	3.53	2.56	88.00	4.60	2.22
Undetected	19.60	0.02	0.01	0.17	0.01	0.00	12.50	0.02	0.00
	ARS			HMA			YS		
Asymptotic	48.00	9.35	5.16	31.00	8.32	5.50	24.50	5.43	3.46
Empirical	48.00	9.32	5.16	28.00	8.29	5.49	20.00	5.39	3.46
Undetected	0.00	0.03	0.00	3.00	0.03	0.01	4.50	0.04	0.00
	SPK			BTM			XSBN		
Asymptotic	34.45	2.55	2.11	54.67	10.86	6.04	27.00	6.25	3.69
Empirical	30.00	2.52	2.11	52.00	10.84	6.04	24.00	6.20	3.68
Undetected	4.45	0.03	0.00	2.67	0.02	0.00	3.00	0.05	0.01
	CBS			TTS			JFL		
Asymptotic	54.00	6.10	4.00	26.50	2.12	1.39	34.25	3.49	2.32
Empirical	45.00	6.09	4.00	25.00	2.12	1.39	32.00	3.49	2.32
Undetected	9.00	0.01	0.00	1.50	0.00	0.00	2.25	0.00	0.00
	DLS			BDGS					
Asymptotic	135.12	11.56	7.06	33.00	3.87	2.59			
Empirical	120.00	11.55	7.06	25.00	3.86	2.59			
Undetected	15.12	0.01	0.00	8.00	0.01	0.00			

Table S4. The iNEXT numerical values for soil fauna of non-asymptotic coverage-based rarefaction and extrapolation and evenness among orders abundances in East Asia.

Step 3. Non-asymptotic coverage-based rarefaction and extrapolation (panel d) Maximum standardized coverage (C_{max})				
Diversity	C_{max}	q=0	q=1	q=2
TH	0.98	71.00	7.89	5.38
ARS	1	48.00	9.32	5.16
SPK	0.96	52.03	2.53	2.11
CBS	1	49.38	6.10	4.00
DLS	1	127.82	11.56	7.06
GH	1	27.17	3.54	2.56
HMA	1	29.90	8.31	5.50
BTM	1	54.07	10.85	6.04
TTS	1	26.30	2.12	1.39
BDGS	1	28.15	3.87	2.59
SMT	1	96.73	4.61	2.22
YS	0.99	22.19	5.42	3.46
XSBN	0.98	25.90	6.23	3.69
JFL	0.96	33.66	3.49	2.32

Step 4: Evenness among species abundances			
Evenness	Pielou *J'*	q=1	q=2
TH	0.48	0.10	0.06
ARS	0.58	0.18	0.09
SPK	0.23	0.03	0.02
CBS	0.46	0.11	0.06
DLS	0.50	0.08	0.05
GH	0.38	0.10	0.06
HMA	0.62	0.25	0.16
BTM	0.60	0.19	0.09
TTS	0.23	0.04	0.02
BDGS	0.41	0.11	0.06
SMT	0.33	0.04	0.01
YS	0.55	0.21	0.12
XSBN	0.56	0.21	0.11
JFL	0.36	0.08	0.04

Table S5. The RDA values for all soil fauna orders in East Asia.

Order Name	Code	RDA1	RDA2	RDA3	RDA4	RDA5	RDA6
Acarina	Aca	-0.0448	0.0220	-0.0089	0.0072	0.0058	0.0016
Amphipoda	Amp	-0.1202	-0.1068	-0.0555	0.0095	-0.0588	-0.0132
Araneae	Ara	-0.0448	0.0220	-0.0089	0.0072	0.0058	0.0016
Archaeogastropoda	Arc	-0.0401	0.0094	0.0764	-0.0761	0.0008	-0.0207
Blattaria	Bla	0.1678	0.0318	-0.0506	0.0185	-0.0418	-0.0692
Chordeumatida	Cho	-0.0667	-0.1331	-0.0699	-0.0431	-0.0369	-0.0012
Coleoptera	Cole	-0.0448	0.0220	-0.0089	0.0072	0.0058	0.0016
Collembola	Coll	-0.0448	0.0220	-0.0089	0.0072	0.0058	0.0016
Dermaptera	Der	0.0697	-0.1139	-0.0811	0.0344	0.0465	-0.0423
Diplura	Dipl	0.1352	-0.0039	0.0829	-0.0368	0.0659	0.0050
Diptera	Dipt	-0.0448	0.0220	-0.0089	0.0072	0.0058	0.0016
Eosentomata	Eos	-0.0282	-0.0531	0.1126	-0.0171	0.0039	0.0157
Geophilomorpha	Geo	-0.0448	0.0220	-0.0089	0.0072	0.0058	0.0016
Glomerida	Glo	0.0515	-0.1117	0.0915	0.0307	0.0182	0.0344
Harpacticoida	Har	0.0915	0.0156	-0.0492	-0.0090	-0.0280	0.0189
Hemiptera	Hem	-0.0811	0.0136	-0.0375	0.0076	-0.0559	0.0507
Homoptera	Hom	-0.0768	0.0599	-0.1251	0.0780	-0.0307	0.0020
Hymenoptera	Hym	-0.0448	0.0220	-0.0089	0.0072	0.0058	0.0016
Isopoda	Isopo	0.1429	-0.0790	0.0887	-0.0264	-0.0845	-0.0063
Isoptera	Isopt	0.1854	0.0064	-0.0129	0.0079	-0.0649	-0.0422
Julida	Julid	0.0033	-0.0576	0.0268	-0.0279	-0.0466	0.0472
Juliformia	Julif	0.0083	0.1580	-0.0117	0.0028	-0.0124	-0.0125
Lepidoptera	Lep	-0.0448	0.0220	-0.0089	0.0072	0.0058	0.0016
Lithobiomorpha	Lit	0.0252	0.0975	0.0877	0.0224	-0.0040	0.0500
Lumbricida	Lum	-0.1923	-0.1262	0.0615	0.0123	-0.0468	-0.0190
Mesogastropoda	Mes	0.0261	-0.2056	-0.0498	-0.0443	-0.0443	0.0030
Mesostigmata	Mesos	-0.0448	0.0220	-0.0089	0.0072	0.0058	0.0016
Microcoryphia	Mic	0.0915	0.0156	-0.0492	-0.0090	-0.0280	0.0189
Neuroptera	Neu	0.0340	-0.0863	0.0538	0.0413	0.0413	0.0075
Opiliones	Opi	0.0154	-0.0402	0.0723	0.1339	-0.0010	0.0010
Orthoptera	Ort	0.0838	-0.0315	-0.0625	-0.0263	0.0760	-0.0403
Plecoptera	Ple	0.0588	0.0138	-0.0337	-0.0425	-0.0487	-0.0033
Polydesmida	Polyd	0.0253	-0.0815	0.0444	-0.0455	-0.0084	0.0183
Polyxenida	Polyx	-0.0144	0.0397	0.0798	-0.0282	-0.0252	-0.0578
Protura	Pro	0.1319	0.0657	-0.0372	0.0307	-0.0062	0.0277
Pseudoscorpiones	Pse	0.0652	-0.0793	-0.0137	-0.0520	0.0756	-0.0434
Psocoptera	Pso	0.1797	0.0023	-0.0531	0.0477	0.0420	0.0490
Rhabditida	Rha	-0.0265	0.1757	0.0148	-0.0924	-0.0015	0.0317
Schizomida	Sch	0.0588	0.0138	-0.0337	-0.0425	-0.0487	-0.0033
Scolopendromorpha	Sco	0.1052	-0.0137	0.0104	-0.1631	0.0190	0.0764
Scutigeromorpha	Scu	0.1065	0.0202	-0.0126	-0.0123	-0.0365	-0.0693

Sinentomata	Sin	-0.0401	0.0094	0.0764	-0.0761	0.0008	-0.0207
Sphaerotheriida	Sph	0.0362	0.0084	0.0285	-0.0004	0.0617	-0.0491
Spirobolida	Spi	0.0482	-0.0541	0.0647	0.0585	0.0648	-0.0128
Stylommatophora	Sty	0.0120	-0.0625	0.0362	0.0590	0.0031	0.0363
Symphyla	Sym	0.1438	0.0032	-0.0010	0.0897	-0.0031	0.0640
Tetramerocerata	Tet	0.1597	-0.0239	-0.0164	-0.0400	-0.0389	-0.0051
Thysanoptera	Thy	0.0252	0.0645	0.1264	0.0521	-0.0846	-0.0319
Thysanura	Thysanura	-0.0411	-0.0699	-0.1051	-0.0249	0.0178	0.0436
Tubificida	Tub	-0.1064	0.0476	-0.0237	-0.0464	0.0576	-0.0307

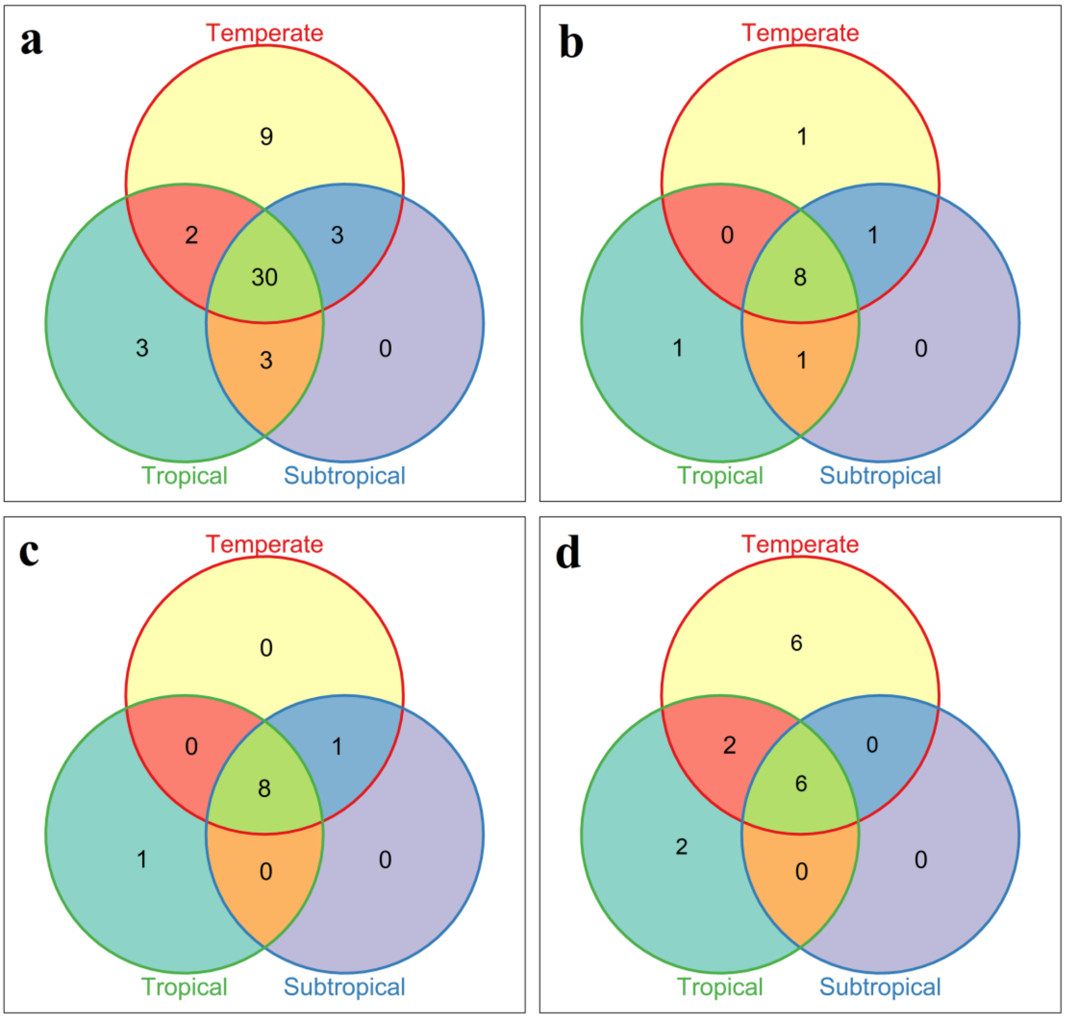

Figure S1. Number of soil fauna orders in temperate, subtropical, and tropical zone of East Asia. The a, b, c and d present the overall, phytophage, predacity, and saprophage soil fauna, respectively.

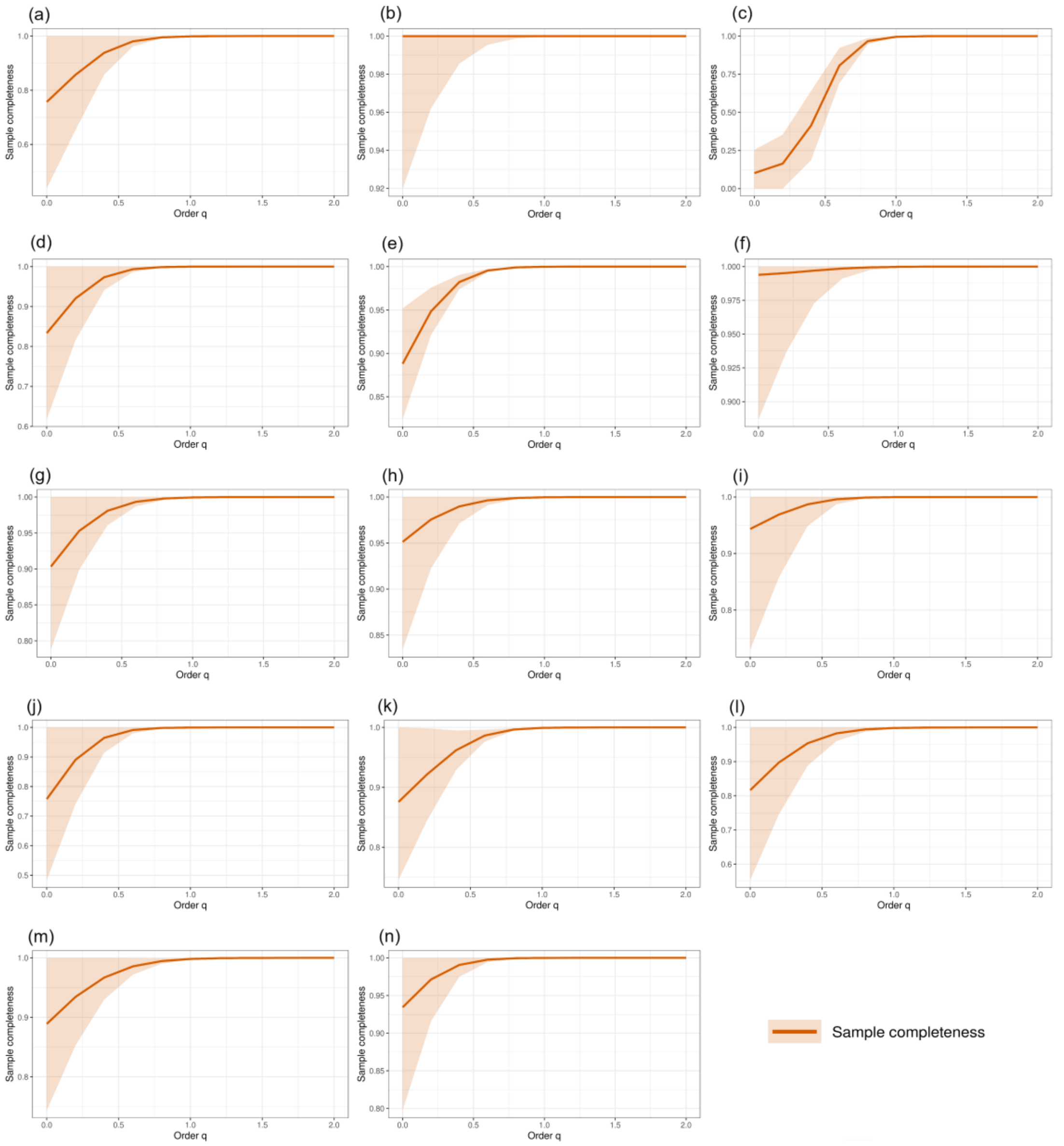

Figure S2. The plots of estimated sample completeness curves as a function of order q between 0 and 2 for soil fauna in fourteen sites at East Asia. Panels (a)-(n) represent Tahe, Aershan, Sapporo, Changbaishan, Donglingshan, Guanghua, Hiroshima, Baotianman, Tinatongshan, Badagongshan, Shimentai, Yushan, Xishuangbanna, and Jianfengling, respectively.

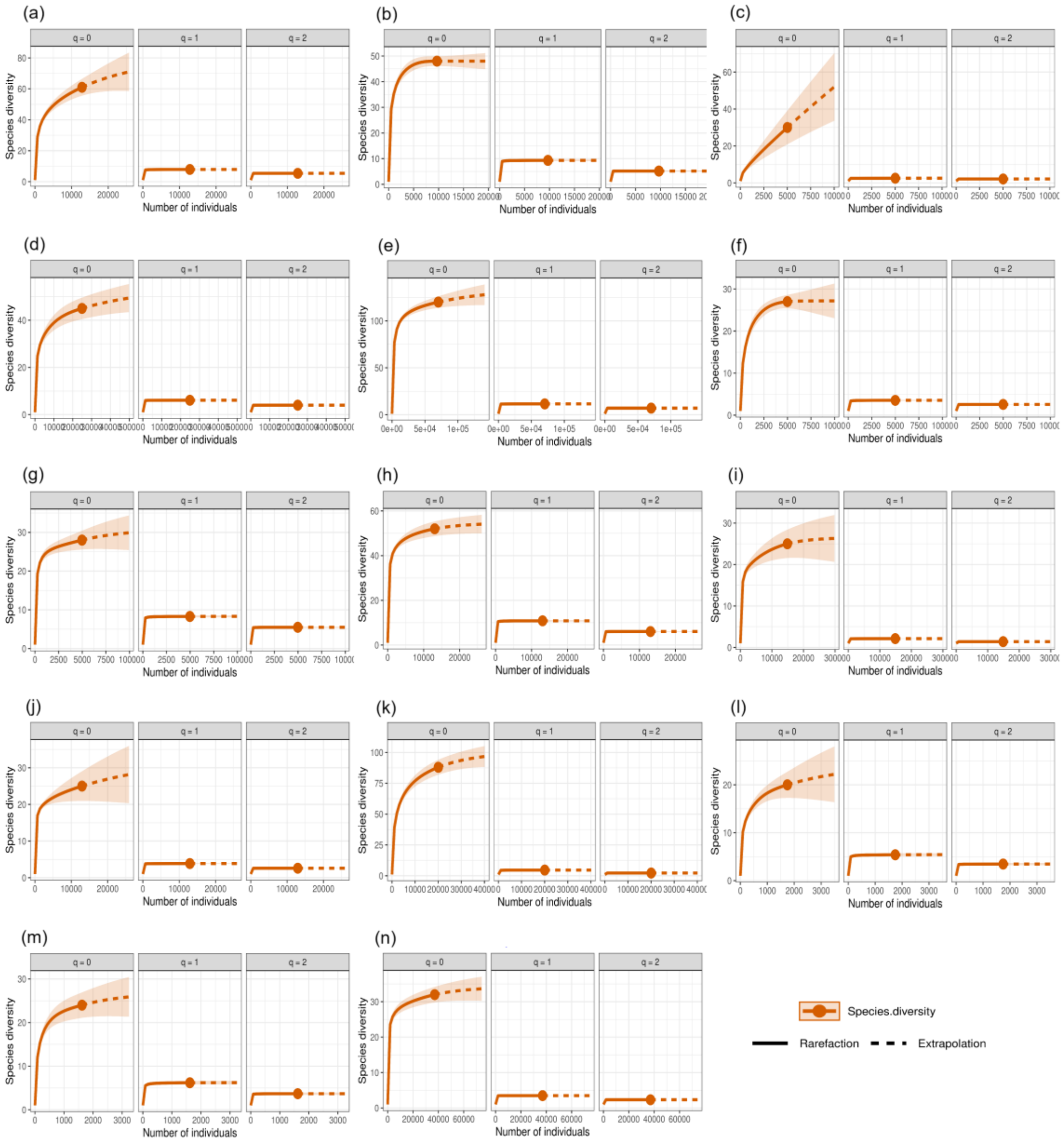

Figure S3. Sample size-based rarefaction (solid lines) and extrapolation curves (dashed lines) for soil fauna in fourteen sites at East Asia. Panels (a)-(n) represent Tahe, Aershan, Sapporo, Changbaishan, Donglingshan, Guanghua, Hiroshima, Baotianman, Tinatongshan, Badagongshan, Shimentai, Yushan, Xishuangbanna, and Jianfengling, respectively.
